# Peripherally Inserted Central Catheter Placement in a Cardiology Ward: A Focus Group Study of Nurses’ Perspectives

**DOI:** 10.3390/ijerph18147618

**Published:** 2021-07-17

**Authors:** Anabela Salgueiro-Oliveira, Rafael A. Bernardes, David Adriano, Beatriz Serambeque, Paulo Santos-Costa, Liliana B. Sousa, Fernando Gama, Rita Barroca, Luciene M. Braga, João Graveto, Pedro Parreira

**Affiliations:** 1 The Health Sciences Research Unit: Nursing (UICISA: E), Nursing School of Coimbra (ESEnfC), 3004-011 Coimbra, Portugal; anabela@esenfc.pt (A.S.-O.); beatrizprazserambeque@esenfc.pt (B.S.); paulocosta@esenfc.pt (P.S.-C.); baptliliana@esenfc.pt (L.B.S.); jgraveto@esenfc.pt (J.G.); parreira@esenfc.pt (P.P.); 2 Coimbra Hospital and Universitary Centre—General Hospital, 3041-801 Coimbra, Portugal; dapa1986@gmail.com; 3 Coimbra Institute for Clinical and Biomedical Research (iCBR) Area of Environment Genetics and Oncobiology (CIMAGO), University of Coimbra, 3000-548 Coimbra, Portugal; 4 Biophysics Institute of Faculty of Medicine, University of Coimbra, 3000-548 Coimbra, Portugal; 5 Center for Innovative Biomedicine and Biotechnology (CIBB), 3000-548 Coimbra, Portugal; 6 Coimbra Hospital and Universitary Centre, 3004-561 Coimbra, Portugal; gama.fj@gmail.com; 7 Hospital dos Lusíadas, 1500-458 Lisbon, Portugal; ritabarroca@hotmail.com; 8 Nursing Department, Federal University of Viçosa, Viçosa 36570-900, Brazil; luciene@daskaleas.com

**Keywords:** catheterization, focus groups, nurse, peripheral

## Abstract

Intravenous therapy administration through peripheral venous catheters is one of the most common nursing procedures performed in clinical contexts. However, peripherally inserted central catheters (PICC) remain insufficiently used by nurses and can be considered a potential alternative for patients who need aggressive intravenous therapy and/or therapy for extended periods. The purpose of this study was to understand nurses’ perspectives about PICC implementation in their clinical practice. As part of an action-research project, three focus groups were developed in June 2019 with nineteen nurses of a cardiology ward from a Portuguese tertiary hospital. From the content analysis, two main categories emerged: ‘nursing practices’ and ‘patients’. Nurses considered PICC beneficial for their clinical practice because it facilitates maintenance care and catheter replacement rates. Moreover, nurses suggested that, since there is a need for specific skills, the constitution of vascular access teams, as recommended by international guidelines, could be an advantage. Regarding patient benefits, nurses highlighted a decrease in the number of venipunctures and also of patient discomfort, which was associated with the number of peripheral venous catheters. Infection prevention was also indicated. As an emerging medical device used among clinicians, peripherally inserted central catheters seem to be essential to clinical practice.

## 1. Introduction

Intravenous (IV) therapy is often performed in hospital settings, mainly through peripheral venous catheters (PVC) [1,2,3,4]. Despite being the most used IV devices, the procedure for its insertion entails associated risks [5]. Additionally, central venous catheters (CVC) are often used when patients have limited peripheral access, when long-term IV medication is needed, as well as for blood product infusions or frequent blood draws [6].

Within the CVC devices, peripherally inserted central catheters (PICC) are claiming more attention from the scientific community. They are intravenous devices inserted through a peripheral superficial vein and introduced until the distal third part of the superior vena cava [7,8].

When compared to traditional CVC, PICC offer advantages such as safer insertion, cost-effective, and convenient placement via specialized nursing teams [9,10]. These devices also avoid multiple venipunctures and allow for the prevention of the main PVC and CVC-related complications [7,8,11,12]. In fact, since PICC requires less repositioning, thus increasing therapy success and patient comfort, some studies state that their cost-effectiveness is greatly increased when compared to other IV devices [13,14].

Therefore, when longer treatments are needed, PICC may be a viable alternative to PVC and to CVC [15,16].

Currently, a multidisciplinary team, including nurses and physicians, is usually in charge of inserting these devices, implying a complex procedure with several interdependent factors, such as patient status and proper vein and catheter selection [17,18]. In recent years, while physicians usually place CVC, nurses have been increasingly assuming the role of inserting PICC and midlines [19], being one of the most important healthcare professionals within specialized vascular access teams. Previously, some studies already verified low bloodstream infection rates, catheter-related thrombosis, or occlusion when PICC were inserted and maintained by an expert nursing team [20].

Even though PICC have many benefits for nursing practice and patient well-being, their usage is uncommon in some clinical contexts. On one side, the introduction of new medical technologies, just like PICC, is an important challenge to institutions and professionals. Nevertheless, nurses usually display positive attitudes towards the use of new technologies [21], particularly since nursing curricula have been enriched with these topics, throughout the years. On the other side, nurses play an important role in continuing medical device development and improvement. Some authors [22,23] have stressed the need to engage nurse professionals in such innovation processes.

In addition, as stated by a recent systematic review [24], individual and systemic factors have implications for nursing practice, since there is a direct influence on patient safety principles, namely quality–care outcomes, which is a core result of nursing care. The authors of the review conclude that there is a need to conduct more studies to enhance the knowledge of particular measures to improve nurses’ adherence to safety principles and effects on patient safety outcomes.

In Portugal, the placement of PICCs by nursing professionals is not yet an established practice in healthcare units, occasionally being used, when needed, in specific contexts. Therefore, this study aimed to understand nurses’ perspectives on implementing PICC in their clinical practice, namely PICC-related advantages and disadvantages and patient-related positive healthcare outcomes. Considering that in this clinical context, PVC was usually used in patients who needed antibiotics for long periods, leading to complications and difficulties for nurses in being able to establish new venous accesses.

## 2. Materials and Methods

### 2.1. Study Design and Setting

As part of an action-research project, which aimed to transfer technological innovations into nursing practice, three focus groups were developed in June 2019 with the nursing team of a cardiology ward from a Portuguese tertiary hospital to explore the potential benefits of using PICC in patients requiring IV antibiotics for long periods compared to PVC. These focus groups were held after a pilot study focusing on PICC implementation in the same nursing ward. Before the pilot study, all nurses had an educational session about PICC insertion and maintenance techniques with ultrasound support. Since the educational session did not consider practical sessions with certification, PICC (Vygon, ref. 801992214V, Lot. 281117VC) were inserted by a certified nurse, who integrated the project.

This first experience motivated the development of focus groups to answer the following research question: «What are the nurses’ perspectives on PICC implementation in their clinical practices?»

### 2.2. Participants

The nursing team (*n* = 26) involved in the pilot study, consisting of the nurses who worked during the period of the pilot study, who had contact with patients with PICC, whether in administering therapy, collecting blood, or involved in its maintenance care, was personally invited by the lead researcher of the project to participate in the focus groups. The schedule of each focus group session was defined taking into consideration the possibility of the entire nursing team being present. Due to unexpected professional and/or personal issues, the convenience sample included nineteen nurses. Participants were allocated in three groups, according to their availability. Six nurses participated in Focus Group 1, six in Focus Group 2, and seven in Focus Group 3. Sociodemographic characteristics were similar between groups. Specifically, eleven nurses (57.9%) were female, with a median age of 42.8 years (SD = 9.7; Min = 31; Max = 61). The majority had a bachelor’s degree, and three (15.8%) had a master’s degree. The average professional experience was 19.6 years (SD = 8.4; Min = 8; Max = 35), of which 12.1 (SD = 9.1; Min = 2; Max = 32) were in the cardiology ward.

### 2.3. Data Collection

The focus groups were carried out on 4th, 12th, and 17th of June, 2019 in a hospital room, and were audio-recorded. They had a 60-min duration each and were moderated by three members of the research team. The interview script was elaborated by the research team (see Supplementary Materials S1: Focus Group Guide) and included questions related to: (a) PICC advantages and disadvantages, both for nursing practice and patient well-being, and (b) possible limitations of introducing the device in the care unit. Information about participants was collected in an earlier project phase.

One group moderator started the meeting with an introduction. The project goals were also described, and permission to record was requested. Active discussion and reflection progressed between nurses, with the moderator ensuring that there were no deviations from the main topic and making sure all participants contributed. By the end, one moderator summarized their perceived content about the discussion and sought to clarify potential misunderstandings. The moderators who conducted the focus groups were project researchers, namely the principal investigator and a researcher responsible for pilot studies on PICC, with extensive experience and nurses’ training, as well as in venous catheters.

### 2.4. Data Analysis and Synthesis

Three researchers transcribed the recorded focus groups, on September 2019, using Microsoft Word and analyzed their content following Bardin’s (2013) [25] approach to thematic content analysis. Registry units were arranged into subcategories and categories, through a thematic/categorical content analysis. The categorical system emerged from exploratory and inductive procedures. Category and subcategory validation was confirmed within the research team. The results of this study followed the consolidated criteria for reporting qualitative research (COREQ recommendations) [26]. The content analysis was performed by December 2019.

### 2.5. Ethical Considerations

This study was approved by the Hospital Ethics Committee (no. 115–17) and by the Portuguese Data Protection Authority (authorization no. 14037/2017). Informed consent was obtained from all participants, and all ethical issues were strictly respected. For confidentiality purposes, each nurse was randomly identified with a capital letter and the focus group number.

## 3. Results

The content analysis revealed different subcategories, which were grouped into two main categories concerning the adoption of PICC: ‘nurses’ practices’ and ‘patients’.

### 3.1. Category 1: Nurses’ Practices

This category shows different aspects that influence nurses’ practice, and emerged from the following subcategories: ‘advantages of using PICC’, ‘limitations of PICC usage’, ‘healthcare team’, and ‘specific skills’. First of all, it is possible to say that the experience with PICC that occurred in the pilot study influenced nurses’ practices in different ways. Nurses considered that there were advantages of using PICC for their practices, as illustrated by the following transcriptions:

*‘It facilitates nurses’ time; it’s better than CVC […]’*

*(O3)*.

*‘[…] it is optimal for blood collection, for people who are only given drugs as bolus […]’*

*(L2)*.

*‘[…] They were beneficial. Catheter handling when the dressing needs replacement […] Catheter indwell time is also an advantage […]’*

*(I2)*.

Limitations of PICC usage for nursing practices were also indicated, particularly the fact that the PICC in use had a single lumen:

*‘It has a single lumen. Several patients receive different drugs, which hinders the procedure […]’*

*(L2)*.

Another subcategory that emerged from the content analysis relates to specific skills for PICC insertion, which were recognized by nurses as essential. Participants recognize that the amount of required training does not justify that all nurses become experts in the procedure:

*‘[…] it isn’t a technique that everyone can develop […]’*

*(S3)*.

*‘In a [healthcare] team there should be nurses with specific skills in this scope’*

*(D2)*.

Since few patients in their care unit met the eligibility criteria for inserting this type of catheter, participants considered that training possibilities are fewer compared to PVC:

*‘[…] the unit casuistry isn’t enough to justify that all nurses have experience in this area […]’*

*(J2)*.

*‘[…] considering the required training for this type of technology, it needs to be divided between all team members […] nobody acquires this enough training […]’*

*(B1)*.

Another identified subcategory was ‘healthcare team’. Participants of the three focus groups agreed that the decision of inserting a PICC is always a responsibility of the whole healthcare team:

*‘[…] the team decides on the PICC insertion […]’*

*(I2)*.

It was also highlighted that some nurses in the hospital should possess additional skills in this area, then be summoned to attend the services, when it was necessary to insert a PICC:

*‘The institution should appoint a professional to move between the different nursing wards […]’*

*(J2)*.

### 3.2. Category 2: Patients

Patients’ category is related to the nurses’ perspective on the adequacy of using PICC in patients of that healthcare unit, as evidenced on the following subcategories: ‘positive healthcare outcomes’ and ‘indications for PICC usage’. Through the experience of inserting a PICC from the pilot study, nurses recognized that there are many healthcare positive outcomes for the patient, namely the reduced number of venipunctures, thus generating less discomfort:

*‘[…] the patient doesn’t have to be submitted to continuous catheterization […] it’s comfortable for the patient’*

*(F1)*.

*‘[…] some patients with six weeks of antibiotherapy are catheterized every 8 hours’*

*(B1)*.

*‘It is great for blood collection in patients with difficult venous access’*

*(L2)*.

The reduction of catheter-related complications is also an advantage compared to CVC:

*‘[…] the infection risk and related complications are much lower […]’*

*(J2)*.

*‘It’s more advantageous than the CVC, in terms of hemorrhage, pneumothorax, infection risk […]’*

*(J2)*.

Lastly, the indications for PICC usage are emphasized for patients with difficult venous access or with specific pathologies, which require aggressive therapy for peripheral veins:

*‘[…] in the case of endocarditis, which often requires 4 to 6 days of continuous antibiotherapy […] the insertion of a PICC makes sense […]’*

*(B1)*.

## 4. Discussion

The participation of the nineteen nurses in the focus groups allowed to elicit the potential positive impact of PICC in nursing practice and on patient’s well-being. This result was confirmed by other studies, which found an improved quality of care in patients requiring a venous catheter [16,27,28].

The topic of specific skills for PICC handling, insertion, and maintenance was much discussed. Nurses considered that training was fundamental for the adoption of this medical device, as addressed in previous studies with nurses [29]. As fewer patients needed a PICC than those needing a PVC in the study setting, its incorporation into nursing practices was a limitation. In another study, nurses identified the same difficulty, that is, few patients being eligible for the insertion of PICC, which otherwise would allow for procedure training [16].

In fact, in a recent study [6], authors concluded that skills related to venipuncture, dressing placement, and other skills were considered easier for nurses when compared to the use of ‘ultrasound’ and placement confirmation devices, which were more difficult to approach and included in daily practice.

In the healthcare sector, many new technologies have become increasingly available [30] to maximize positive treatment outcomes and efficiency. However, the implementation of new technologies that require a modification of nursing practices may hamper their use [31]. The implementation of new technologies into practice is not a simple process [32] primarily due to routine changes and long periods of adaptation.

In our study, nurses considered that the institution needs to create a vascular access team dedicated to PICC insertion, to speed up procedures and bring comfort to patients and their families. Interestingly, a recent study with 140 nurses showed that the majority of PICC insertions occurred within the institutions [9], denoting some degree of support. In this sense, there might be strong institutional and organizational influences and disparities among countries, which is also seen in how nurses adopt, or not, specific guidelines [33]. Innovative measures, as the introduction of new technologies in already established procedures, demand actions not only by isolated nurses or physicians, since they are part of a wider organization [30]. In this sense, organizational issues were mostly discussed to understand the existence of vascular access teams in hospitals for visits to each nursing ward and specific procedures related to PICC insertion. This topic is described in international recommendations for safe, effective, and high-quality infusion therapy [34,35,36].

Considering the cardiology ward where the study was developed, nurses considered the single-lumen PICCs a disadvantage, limiting its use or actual future implementation. Although PICC are available with different structures [7,37], including with more than one lumen, the one used in the study was considered a significant limitation. Despite this conclusion, the literature states that single-lumen PICCs reduced vessel thrombosis and respective risk, when compared to multi-lumen [38]. In fact, these devices allow for the administration of any drug, even the most aggressive and/or vesicant, thus being a good PVC alternative [16,39]

Although nurses identified some negative aspects, one of the most advantageous characteristics was that PICC contributed to the reduction of the number of venipunctures, patient discomfort, and related complications in inpatients with endocarditis and requiring extended periods of antibiotherapy, similarly to some studies [40]. Of the complications that could be mitigated, infection was the most indicated by nurses, since the PICC allows for a better adaptation to the patient’s skin, prevents repetitive catheterizations, and unnecessary site handling. Catheter-related infections, as bloodstream infections, which can result in worse complications like sepsis, are a burden to hospitals and should be eliminated, as they generate hospital expenses, work-related stress, and patient and family discomfort [41,42].

This study had some limitations, such as the availability of just two single-lumen PICC, which decreased the diversity of experiences with the device.

## 5. Conclusions

PICC, as a medical device used among clinicians, particularly nurses and physicians, seem to be an essential device to be used in hospitals. Nurses consider that its incorporation into practice might decrease infection, increase patients’ quality of life, and optimize some procedures. However, nurses are aware that this device requires specific skills, which should be promoted by the healthcare institutions, through investment in the specific training of nurses on PICC insertion and maintenance for the skills development, as well as the constitution of dedicated vascular access teams.

The selection of PICC has well-defined criteria, leading to a limited number of eligible patients. Thus, the possibility of healthcare professionals training in each care unit is restricted, narrowing the development of skills. More studies are needed to understand the most relevant aspects about PICC handling guidelines and professionals’ skills, its impact on clinical practice, and cost-effectiveness compared to PVC. Healthcare professionals, namely nurses, should be asked about their daily clinical practices, for a more efficient PICC incorporation into daily routines.

## Data Availability

The data presented in this study are available on request from the corresponding author. The data are not publicly available due to ethical considerations, regarding personal information and to respect what was written in the signed informed consent.

## Supplementary Materials

The following are available online at https://www.mdpi.com/article/10.3390/ijerph18147618/s1, S1: Focus Group Guide.
