# Supplementary material for: Peripherally Inserted Central Catheter Placement in a Cardiology Ward: A Focus Group Study of Nurses’ Perspectives
Source: Int J Environ Res Public Health. 2021 Jul 17;18(14):7618. doi: 10.3390/ijerph18147618 (PMC8303562; doi:10.3390/ijerph18147618)
Supplement: Supplementary file 1 [file ijerph-18-07618-s001.zip › ijerph-1283713-supplementary.pdf]

Focus Group Guide

Interviewer: Date: Time:
Moderator:
Location:

Introduction	Comments
1. Brief overview of the TecPrevInf project.	- Consider the current reflection stage of this project.
2. Description of the Focus Group, addressing:<br>a) Its objectives;<br>b) The role of the researcher and moderator during the Focus Group;<br>c) Its structure;<br>d) Data processing;<br>e) Confidentiality.	
3. Permission request to use audio recording for the discussion.	- Request permission.
4. Development	
5. Objectives:<br>a) To establish the changes in nurses' practices after the introduction of peripherally inserted central venous catheters (PICCs) in the healthcare unit.<br>b) To determine the advantages and disadvantages of PICCs.<br>c) To identify the factors that hindered or promoted the use of PICCs.<br>d) To understand the perception of the nursing team on adherence to the PICC medical device.<br>e) To establish the relevant actions that should be implemented to improve PICC adherence.	- Contextualise within the TecPrevInf project.
6. Questions:<br>a) Which advantages and disadvantages does the PICC technology provide to patients and the nursing team?<br>b) What factors may have influenced the nursing team to adhere to PICCs?<br>c) Which relevant efforts should be implemented to encourage the nursing team to adhere to PICCs and improve nurses' practices in this area?	Approach:<br>- Develop the questions to suit the characteristics of the group.<br>- Emit verbal and non-verbal signals of attention and understanding.<br>- Ask for clarifications on understanding the phenomenon.<br>- Help with follow-up questions, if necessary.<br>- Add other pertinent questions, bearing in mind the objective

	and based on what is reported.<br>- Allow everyone to participate in the focus group.
7. Closing:<br>a) Summarize the discussed topics.<br>b) Allow the group to add or explain some point.	- Thank the group for their trust and cooperation.
